# Supplementary material for: Racial Differences in Vaginal Fluid Metabolites and Association with Systemic Inflammation Markers among Ovarian Cancer Patients: A Pilot Study
Source: Cancers (Basel). 2024 Mar 23;16(7):1259. doi: 10.3390/cancers16071259 (PMC11011195; doi:10.3390/cancers16071259)
Supplement: Supplementary file 1 [file cancers-16-01259-s001.zip › cancers-2897113-supplementary.pdf]

Supplementary material for "Racial differences in vaginal fluid metabolites and association with systemic inflammation markers among ovarian cancer patients: A pilot study"

Table S1. Spearman's rank correlation coefficient (ρ) between metabolites and inflammation biomarkers and corresponding *P*-values for overall study cohort and by race; also showing Z-score and *P*-values from Fisher's *Z* test comparing correlation coefficients between Black vs White groups.

Metabolite	Overall: ρ	FDR	Black: ρ	Black: *P*-value	White: ρ	White: *P* -value	Z-score	*P* -value	Biomarker
C14:1	0.47	0.023	0.61	0.065	0.49	0.051	0.47	0.647	IL1
C14-OH/C12-DC	0.57	0.004	0.46	0.123	0.63	0.018	0.57	0.517	IL1
C16	0.62	0.001	0.6	0.065	0.63	0.018	0.62	0.898	IL1
C16:1	0.59	0.002	0.65	0.055	0.56	0.041	0.59	0.706	IL1
C16:1-OH/C14:1-DC	0.43	0.035	0.47	0.123	0.45	0.058	0.43	0.946	IL1
C16:2	0.52	0.01	0.39	0.199	0.64	0.018	0.52	0.358	IL1
C16-OH/C14-DC	0.49	0.019	0.26	0.351	0.62	0.018	0.49	0.224	IL1
C18:1-DC	0.64	<0.001	0.82	0.003	0.54	0.042	0.64	0.143	IL1
C18-OH/C16-DC	0.46	0.024	0.16	0.558	0.55	0.041	0.46	0.225	IL1
C20:4	0.43	0.036	0.58	0.065	0.35	0.14	0.43	0.431	IL1
C22	0.72	<0.001	0.82	0.003	0.69	0.018	0.72	0.413	IL1
C5	-0.44	0.033	-0.68	0.045	-0.33	0.15	-0.44	0.197	IL1
Cer(d18:1/16:0)	-0.5	0.015	-0.46	0.123	-0.54	0.042	-0.5	0.777	IL1
SM(d31:0)	0.7	<0.001	0.75	0.02	0.65	0.018	0.7	0.6	IL1
SM(d31:1)	0.7	<0.001	0.72	0.028	0.67	0.018	0.7	0.797	IL1
SM(d33:1)	-0.52	0.01	-0.58	0.065	-0.5	0.051	-0.52	0.764	IL1
SM(d33:3)	-0.42	0.039	-0.3	0.302	-0.5	0.051	-0.42	0.525	IL1
SM(d34:1)	-0.41	0.048	-0.35	0.223	-0.49	0.051	-0.41	0.651	IL1
SM(d35:1)	-0.53	0.01	-0.48	0.123	-0.43	0.066	-0.53	0.867	IL1
SM(d36:1)	-0.48	0.019	-0.59	0.065	-0.45	0.058	-0.48	0.609	IL1
SM(d37:1)	-0.56	0.004	-0.56	0.071	-0.45	0.058	-0.56	0.694	IL1
SM(d38:1)	-0.47	0.023	-0.51	0.105	-0.4	0.09	-0.47	0.712	IL1
SM(d39:1)	-0.59	0.002	-0.6	0.065	-0.55	0.041	-0.59	0.843	IL1
SM(d40:1)	-0.49	0.019	-0.56	0.071	-0.49	0.051	-0.49	0.797	IL1
SM(d41:1)	-0.48	0.02	-0.54	0.084	-0.45	0.058	-0.48	0.751	IL1
SM(d42:1)	-0.46	0.024	-0.46	0.123	-0.51	0.051	-0.46	0.862	IL1
SM(d43:1)	-0.44	0.034	-0.36	0.22	-0.5	0.051	-0.44	0.647	IL1
SM(d44:1)	-0.42	0.041	-0.38	0.199	-0.48	0.053	-0.42	0.744	IL1
SM(d44:2)	-0.41	0.044	-0.38	0.202	-0.46	0.058	-0.41	0.773	IL1
SM(d45:0)	-0.46	0.024	-0.45	0.123	-0.48	0.053	-0.46	0.919	IL1

Table S1. Spearman's rank correlation coefficient (ρ) between metabolites and inflammation biomarkers and corresponding *P*-values for overall study cohort and by race; also showing Z-score and *P*-values from Fisher's *Z* test comparing correlation coefficients between Black vs White groups.

**Metabolite**	**Overall: ρ**	**FDR**	**Black: ρ**	**Black: *P*-value**	**White: ρ**	**White: *P* -value**	**Z-score**	***P* -value**	**Biomarker**
SM(d45:1)	-0.44	0.033	-0.46	0.123	-0.45	0.058	-0.44	0.973	IL1
C12:1	0.42	0.038	0.23	0.45	0.56	0.033	0.42	0.29	IL10
C14:1	0.51	0.013	0.7	0.036	0.49	0.044	0.51	0.38	IL10
C14-OH/C12-DC	0.57	0.004	0.43	0.197	0.62	0.021	0.57	0.482	IL10
C16	0.65	<0.001	0.55	0.136	0.66	0.019	0.65	0.644	IL10
C16:1	0.6	0.002	0.61	0.089	0.61	0.023	0.6	>0.999	IL10
C16:1-OH/C14:1-DC	0.45	0.024	0.45	0.179	0.45	0.053	0.45	>0.999	IL10
C16:2	0.55	0.006	0.42	0.199	0.64	0.021	0.55	0.41	IL10
C16-OH/C14-DC	0.49	0.016	0.35	0.271	0.62	0.021	0.49	0.34	IL10
C18:1-DC	0.67	<0.001	0.79	0.012	0.57	0.033	0.67	0.261	IL10
C18-OH/C16-DC	0.46	0.023	0.18	0.524	0.56	0.033	0.46	0.232	IL10
C20:4	0.45	0.026	0.52	0.136	0.38	0.108	0.45	0.64	IL10
C22	0.75	<0.001	0.79	0.012	0.71	0.019	0.75	0.625	IL10
C5	-0.44	0.029	-0.63	0.083	-0.37	0.111	-0.44	0.349	IL10
Cer(d18:1/16:0)	-0.54	0.007	-0.5	0.145	-0.56	0.033	-0.54	0.825	IL10
GlcCer.d18:1/22:0.	0.4	0.046	0.18	0.524	0.49	0.044	0.4	0.348	IL10
Glycerol	0.41	0.042	0.47	0.177	0.34	0.139	0.41	0.679	IL10
SM(d31:0)	0.72	<0.001	0.74	0.03	0.66	0.019	0.72	0.676	IL10
SM(d31:1)	0.72	<0.001	0.7	0.036	0.68	0.019	0.72	0.919	IL10
SM(d33:1)	-0.53	0.007	-0.52	0.136	-0.51	0.037	-0.53	0.971	IL10
SM(d33:3)	-0.45	0.024	-0.21	0.486	-0.55	0.035	-0.45	0.282	IL10
SM(d34:1)	-0.44	0.028	-0.29	0.382	-0.52	0.037	-0.44	0.461	IL10
SM(d35:1)	-0.54	0.007	-0.41	0.201	-0.44	0.062	-0.54	0.923	IL10
SM(d36:1)	-0.5	0.014	-0.52	0.136	-0.47	0.05	-0.5	0.86	IL10
SM(d37:1)	-0.58	0.003	-0.52	0.136	-0.47	0.05	-0.58	0.86	IL10
SM(d38:1)	-0.49	0.017	-0.46	0.177	-0.42	0.075	-0.49	0.895	IL10
SM(d38:2)	-0.41	0.044	-0.25	0.421	-0.53	0.037	-0.41	0.375	IL10
SM(d39:1)	-0.61	0.002	-0.56	0.136	-0.57	0.033	-0.61	0.969	IL10
SM(d40:1)	-0.51	0.012	-0.5	0.145	-0.52	0.037	-0.51	0.943	IL10
SM(d41:1)	-0.48	0.018	-0.45	0.179	-0.46	0.051	-0.48	0.973	IL10

Table S1. Spearman's rank correlation coefficient (ρ) between metabolites and inflammation biomarkers and corresponding *P*-values for overall study cohort and by race; also showing Z-score and *P*-values from Fisher's *Z* test comparing correlation coefficients between Black vs White groups.

Metabolite	Overall: ρ	FDR	Black: ρ	Black: *P*-value	White: ρ	White: *P* -value	Z-score	*P* -value	Biomarker
SM(d42:1)	-0.47	0.02	-0.36	0.268	-0.53	0.037	-0.47	0.572	IL10
SM(d43:1)	-0.44	0.028	-0.23	0.45	-0.51	0.037	-0.44	0.384	IL10
SM(d44:1)	-0.44	0.029	-0.27	0.417	-0.51	0.037	-0.44	0.448	IL10
SM(d44:2)	-0.42	0.035	-0.26	0.419	-0.49	0.044	-0.42	0.474	IL10
SM(d45:0)	-0.47	0.021	-0.38	0.244	-0.51	0.037	-0.47	0.666	IL10
SM(d45:1)	-0.45	0.024	-0.37	0.253	-0.48	0.045	-0.45	0.721	IL10
C12:1	0.43	0.03	0.26	0.39	0.57	0.03	0.43	0.312	TNF
C14:1	0.52	0.009	0.65	0.062	0.51	0.034	0.52	0.573	TNF
C14-OH/C12-DC	0.56	0.004	0.48	0.163	0.6	0.026	0.56	0.652	TNF
C16	0.66	<0.001	0.59	0.101	0.68	0.018	0.66	0.688	TNF
C16:1	0.61	0.002	0.66	0.056	0.6	0.026	0.61	0.792	TNF
C16:1-OH/C14:1-DC	0.48	0.015	0.47	0.166	0.49	0.039	0.48	0.945	TNF
C16:2	0.53	0.007	0.39	0.238	0.63	0.025	0.53	0.382	TNF
C16-OH/C14-DC	0.5	0.011	0.31	0.326	0.65	0.018	0.5	0.228	TNF
C18:1-DC	0.68	<0.001	0.83	0.004	0.59	0.026	0.68	0.176	TNF
C18-OH/C16-DC	0.49	0.014	0.16	0.597	0.6	0.026	0.49	0.158	TNF
C20:4	0.46	0.019	0.57	0.101	0.39	0.098	0.46	0.532	TNF
C22	0.76	<0.001	0.83	0.004	0.72	0.017	0.76	0.457	TNF
C5	-0.42	0.035	-0.67	0.056	-0.33	0.15	-0.42	0.215	TNF
Cer(d18:1/16:0)	-0.56	0.004	-0.46	0.173	-0.59	0.026	-0.56	0.632	TNF
GlcCer.d18:1/22:0.	0.43	0.029	0.23	0.45	0.53	0.03	0.43	0.345	TNF
Glycerol	0.4	0.043	0.47	0.166	0.34	0.15	0.4	0.679	TNF
SM(d31:0)	0.73	<0.001	0.76	0.021	0.66	0.018	0.73	0.59	TNF
SM(d31:1)	0.73	<0.001	0.72	0.035	0.69	0.018	0.73	0.874	TNF
SM(d32:1)	-0.4	0.043	-0.19	0.531	-0.49	0.039	-0.4	0.362	TNF
SM(d33:1)	-0.56	0.004	-0.57	0.101	-0.54	0.03	-0.56	0.908	TNF
SM(d33:3)	-0.47	0.019	-0.29	0.349	-0.54	0.03	-0.47	0.418	TNF
SM(d34:1)	-0.45	0.023	-0.34	0.301	-0.54	0.03	-0.45	0.507	TNF
SM(d34:2)	-0.4	0.043	-0.06	0.832	-0.55	0.03	-0.4	0.139	TNF
SM(d35:1)	-0.55	0.005	-0.44	0.18	-0.46	0.048	-0.55	0.947	TNF

Table S1. Spearman's rank correlation coefficient (ρ) between metabolites and inflammation biomarkers and corresponding *P*-values for overall study cohort and by race; also showing Z-score and *P*-values from Fisher's *Z* test comparing correlation coefficients between Black vs White groups.

**Metabolite**	**Overall: ρ**	**FDR**	**Black: ρ**	**Black: *P*-value**	**White: ρ**	**White: *P* -value**	**Z-score**	***P* -value**	**Biomarker**
SM(d36:1)	-0.5	0.012	-0.56	0.101	-0.47	0.044	-0.5	0.745	TNF
SM(d37:1)	-0.59	0.002	-0.52	0.131	-0.46	0.048	-0.59	0.834	TNF
SM(d38:1)	-0.49	0.014	-0.5	0.152	-0.41	0.078	-0.49	0.763	TNF
SM(d38:2)	-0.41	0.039	-0.3	0.33	-0.53	0.03	-0.41	0.457	TNF
SM(d39:1)	-0.62	<0.001	-0.59	0.101	-0.57	0.03	-0.62	0.936	TNF
SM(d40:1)	-0.52	0.009	-0.55	0.105	-0.52	0.032	-0.52	0.911	TNF
SM(d41:1)	-0.51	0.01	-0.5	0.152	-0.49	0.039	-0.51	0.972	TNF
SM(d41:2)	-0.42	0.034	-0.14	0.637	-0.54	0.03	-0.42	0.219	TNF
SM(d42:1)	-0.49	0.014	-0.44	0.18	-0.54	0.03	-0.49	0.726	TNF
SM(d43:1)	-0.47	0.017	-0.31	0.326	-0.54	0.03	-0.47	0.452	TNF
SM(d43:2)	-0.43	0.03	-0.32	0.319	-0.47	0.045	-0.43	0.636	TNF
SM(d44:1)	-0.45	0.022	-0.35	0.301	-0.53	0.03	-0.45	0.551	TNF
SM(d44:2)	-0.44	0.027	-0.34	0.301	-0.5	0.036	-0.44	0.605	TNF
SM(d45:0)	-0.5	0.013	-0.44	0.18	-0.54	0.03	-0.5	0.726	TNF
SM(d45:1)	-0.49	0.014	-0.42	0.194	-0.52	0.032	-0.49	0.733	TNF
C12:1	0.41	0.039	0.25	0.393	0.54	0.04	0.41	0.355	IL6
C14:1	0.5	0.013	0.64	0.068	0.46	0.055	0.5	0.489	IL6
C14-OH/C12-DC	0.57	0.003	0.47	0.162	0.65	0.02	0.57	0.482	IL6
C16	0.63	<0.001	0.58	0.113	0.63	0.021	0.63	0.834	IL6
C16:1	0.59	0.003	0.65	0.068	0.59	0.035	0.59	0.796	IL6
C16:1-OH/C14:1-DC	0.45	0.026	0.45	0.169	0.43	0.062	0.45	0.948	IL6
C16:2	0.55	0.006	0.38	0.244	0.68	0.018	0.55	0.255	IL6
C16-OH/C14-DC	0.49	0.015	0.34	0.3	0.63	0.021	0.49	0.304	IL6
C18:1-DC	0.67	<0.001	0.82	0.005	0.56	0.04	0.67	0.165	IL6
C18-OH/C16-DC	0.45	0.026	0.15	0.61	0.55	0.04	0.45	0.215	IL6
C20:4	0.45	0.027	0.54	0.116	0.36	0.12	0.45	0.547	IL6
C22	0.74	<0.001	0.82	0.005	0.7	0.018	0.74	0.443	IL6
C5	-0.44	0.03	-0.64	0.068	-0.37	0.115	-0.44	0.327	IL6
Cer(d18:1/16:0)	-0.53	0.008	-0.46	0.162	-0.54	0.04	-0.53	0.777	IL6
GlcCer.d18:1/22:0.	0.42	0.038	0.23	0.435	0.5	0.042	0.42	0.403	IL6

Table S1. Spearman's rank correlation coefficient (ρ) between metabolites and inflammation biomarkers and corresponding *P*-values for overall study cohort and by race; also showing Z-score and *P*-values from Fisher's *Z* test comparing correlation coefficients between Black vs White groups.

Metabolite	Overall: ρ	FDR	Black: ρ	Black: *P*-value	White: ρ	White: *P* -value	Z-score	*P* -value	Biomarker
Glycerol	0.42	0.035	0.48	0.16	0.38	0.106	0.42	0.744	IL6
SM(d31:0)	0.71	<0.001	0.77	0.015	0.65	0.02	0.71	0.516	IL6
SM(d31:1)	0.72	<0.001	0.71	0.035	0.68	0.018	0.72	0.878	IL6
SM(d33:1)	-0.53	0.008	-0.55	0.116	-0.5	0.042	-0.53	0.855	IL6
SM(d33:3)	-0.46	0.025	-0.26	0.381	-0.54	0.04	-0.46	0.37	IL6
SM(d34:1)	-0.43	0.03	-0.32	0.313	-0.51	0.042	-0.43	0.54	IL6
SM(d35:1)	-0.53	0.008	-0.43	0.198	-0.44	0.061	-0.53	0.974	IL6
SM(d36:1)	-0.5	0.013	-0.55	0.116	-0.48	0.05	-0.5	0.8	IL6
SM(d37:1)	-0.58	0.003	-0.52	0.134	-0.46	0.055	-0.58	0.834	IL6
SM(d38:1)	-0.49	0.014	-0.49	0.16	-0.44	0.061	-0.49	0.866	IL6
SM(d38:2)	-0.41	0.039	-0.29	0.345	-0.53	0.04	-0.41	0.439	IL6
SM(d39:1)	-0.6	0.002	-0.58	0.113	-0.55	0.04	-0.6	0.938	IL6
SM(d40:1)	-0.51	0.011	-0.54	0.116	-0.51	0.042	-0.51	0.913	IL6
SM(d41:1)	-0.49	0.015	-0.48	0.16	-0.45	0.058	-0.49	0.919	IL6
SM(d41:2)	-0.41	0.041	-0.13	0.639	-0.51	0.042	-0.41	0.252	IL6
SM(d42:1)	-0.48	0.016	-0.4	0.229	-0.53	0.04	-0.48	0.659	IL6
SM(d43:1)	-0.45	0.026	-0.27	0.381	-0.5	0.042	-0.45	0.47	IL6
SM(d43:2)	-0.41	0.04	-0.31	0.323	-0.44	0.061	-0.41	0.687	IL6
SM(d44:1)	-0.44	0.027	-0.31	0.327	-0.5	0.042	-0.44	0.544	IL6
SM(d44:2)	-0.43	0.03	-0.32	0.313	-0.5	0.042	-0.43	0.564	IL6
SM(d45:0)	-0.47	0.021	-0.4	0.229	-0.48	0.048	-0.47	0.792	IL6
SM(d45:1)	-0.46	0.025	-0.39	0.229	-0.46	0.055	-0.46	0.821	IL6

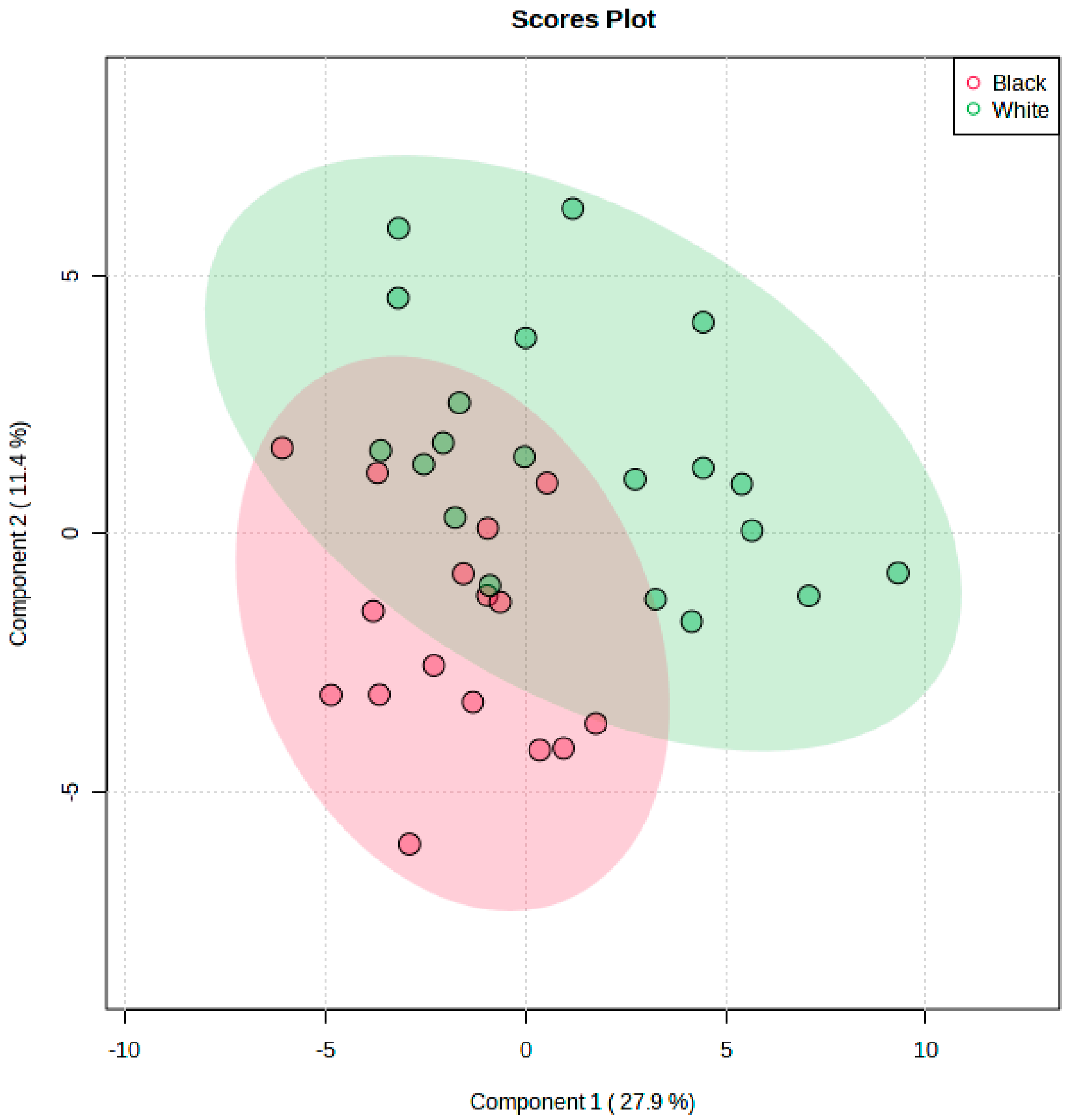

Supplementary Figure S1. 2D scores plot showing PLS-DA overlapping discrimination between vaginal fluid metabolites from cervicovaginal fluid of Black vs White patients.

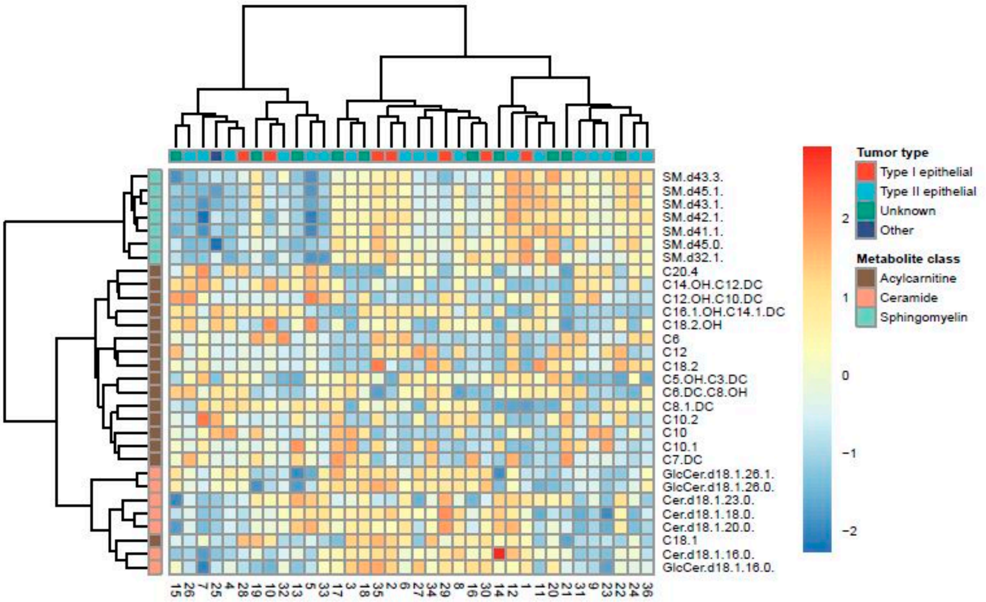

Supplementary Figure S2. Heat map showing hierarchical clusters by OC tumor type, based on top 30 targeted metabolites with the largest variability identified by interquartile range.

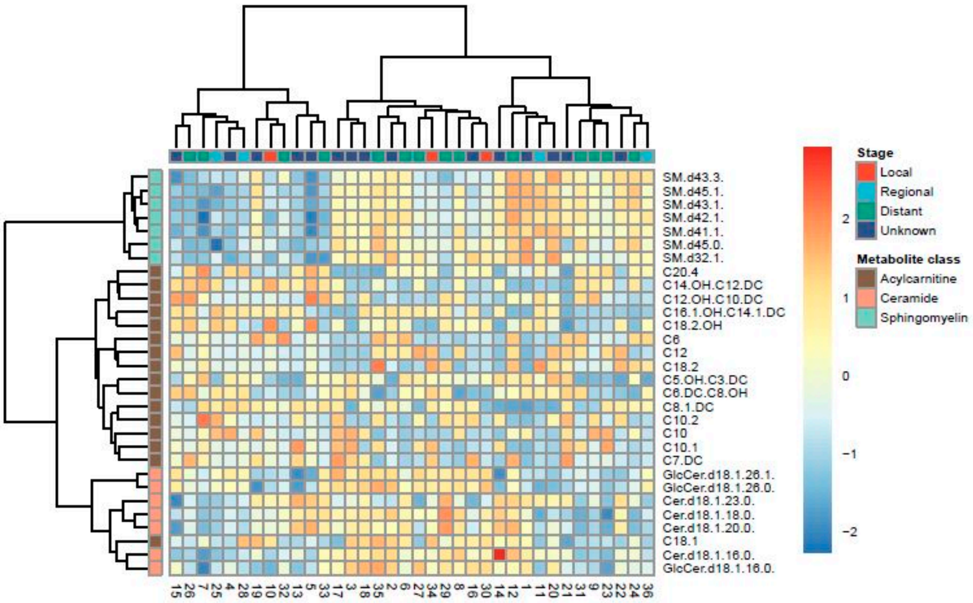

Supplementary Figure S3. Heat map showing hierarchical clusters by OC tumor stage, based on top 30 targeted metabolites with the largest variability identified by interquartile range.
